# Risk of Dislocation After Total Hip Arthroplasty in Patients with Crowe Type IV Developmental Dysplasia of the Hip

**DOI:** 10.1111/os.12665

**Published:** 2020-03-29

**Authors:** Zi‐chuan Ding, Wei‐nan Zeng, Ping Mou, Zhi‐min Liang, Duan Wang, Zong‐ke Zhou

**Affiliations:** ^1^ Department of Orthopaedics West China Hospital/West China School of Medicine, Sichuan University Chengdu China; ^2^ Clinic Research Management Department West China Hospital/West China School of Medicine, Sichuan University Chengdu China

**Keywords:** Developmental dysplasia of the hip, Postoperative dislocation, Total hip arthroplasty

## Abstract

**Objective:**

To investigate whether the risk of dislocation after total hip arthroplasty (THA) in patients with Crowe type IV developmental dysplasia of the hip (DDH) is high and to further identify the risk factors for postoperative dislocation in these patients.

**Methods:**

This retrospective cohort study reviewed Crowe type IV DDH patients undergoing THA between January 2009 and December 2017 in our institution. Each Crowe type IV DDH patient was matched with three Crowe type I, II, or III DDH patients according to gender, side and date of operation. The primary outcome of this study was postoperative dislocation after THA. Occurrence, rate, classification, treatment and outcome of dislocation were documented in detail for all patients. The dislocation rates were compared between Crowe type IV DDH patients and Crowe type I, II, or III DDH patients. Demographic data, implant factors, and surgical factors were compared between the dislocation and no dislocation groups. Multiple logistic regression analysis was used to determine the independent risk factors for dislocation in Crowe type IV hips.

**Results:**

A total of 131 Crowe type IV hips were followed up for a mean of 76.5 ± 28.1 months. Three hundred and ninety‐three Crowe type I, II and III hips, including 261 type I hips, 94 type II hips, and 38 type III hips, were identified as controls and followed up for a mean of 76.4 ± 28.2 months. No significant difference was observed in follow‐up time between two groups (*P* = 0.804). One or more dislocations occurred in 22 of the 524 dysplasia hips (4.20%). Of the 22 dislocated hips, 20 hips (90.9%) were successfully managed with non‐operative treatment. Two patients (9.1%, one Crowe type I and one Crowe type IV) experienced recurrent dislocation and required revision surgery. Crowe type IV hips had a significantly higher postoperative dislocation rate than type I, II, and III hips (11.45% *vs* 1.78%, *P* < 0.001). The use of a 22‐mm femoral head (odds ratio [*OR*] = 23.55, 95% confidence interval [*CI*] = 1.901–291.788, *P* = 0.014), older age (*OR* = 1.128, 95% *CI* = 1.037–1.275, *P* = 0.031), and absence of false acetabulum (*OR* = 12.425, 95% *CI* = 1.982–77.879, *P* = 0.007) were identified as independent risk factors for dislocation in Crowe type IV hips.

**Conclusions:**

Crowe type IV DDH patients were at a high risk of dislocation after THA, and using large femoral heads and improving abductor muscle strength may help decrease the rate of postoperative dislocation in such patients.

## Introduction

Total hip arthroplasty (THA) is considered to be the successful treatment of choice for end‐stage hip diseases, and provides excellent postoperative pain relief, remarkable deformity correction, and satisfactory function recovery^1^. Dislocation after THA is reported to be the most common cause of revision THA, accounting for 17.3% of revision THA cases in the United States^2^. It has been reported that more than 60% of the patients that sustain a postoperative dislocation have recurrence and more than 50% required revision THA^3^. Although much effort has been devoted to its prevention, the incidence of dislocation after primary THA still ranges from 1.4% to 5.0%^4^, ^5^, ^6^. The risk factors for postoperative dislocation identified include patient factors (including advanced age, abductor muscle weakness, preoperative diagnosis, and so on) and surgical factors (including small femoral head size, component malposition and low soft tissue tension, and so on)^7^, ^8^, ^9^. Besides, the surgeon experience can influence the postoperative dislocation rate. A Canadian study involving 38,000 patients found that surgeons who performed over 35 THAs per year had a lower dislocation rate than surgeons with smaller volumes^10^.

The operative principles of THA are restoration of the hip rotation center and recreating the offset and length of lower limb, which restore the soft‐tissue tension around the hip and significantly decrease the rate of postoperative dislocation^11^. Implantation of acetabular component in safe zones also improves the stability of the hip. Lewinnek *et al*. believed a cup position in a “safe zone” of 15 ± 10 degrees of anteversion and 40 ± 10 degrees of inclination can minimize the postoperative dislocation rate^12^. Combined anteversion technique, combining the anteversion of acetabular and femoral component within safe zone of 25 degrees to 50 degrees, has been suggested in recent years^13^. The large femoral head diameter has been widely accepted as a protective factor for dislocation after THA because of reduced component impingement and increased “jump distance”^7^, ^8^. Abductor muscle plays an important part in maintaining the stability of the hip. Causes leading to abductor muscle weakness, such as neuromuscular diseases, compromise of the abductor‐trochanteric complex, and gluteus medius injury, are known to influence the postoperative stability of the hip^14^. Preoperative and postoperative improvement in abductor muscle strength through rehabilitation programs helps prevent postoperative dislocation.

Developmental dysplasia of the hip (DDH) presents developmental bony deformity and soft tissue abnormality, and is a common cause of secondary osteoarthritis of the hip, which eventually requires THA^15^. Based on the subluxation height relative to the interteardrop line, the severity of DDH was divided into four types according to Crowe^16^. Crowe type IV DDH has a high dislocation of the femoral head and is technically difficult to treat by THA because of a wide spectrum of anatomical abnormalities, which are as follows: small and shallow true acetabulum with anterior and superior bone deficiency; femoral deformities with excessive anteversion of the femoral neck; and soft tissue abnormalities including horizontal orientation and weakened abductor muscle as well as hypertrophic capsule^17^, ^18^. The dislocation rate after THA was suspected to be high in the presence of such deformity. Minoda *et al*. found that in Crowe type IV hips, the risk of placing an acetabular component outside the safe range was high^19^.

The dislocation rate after THA in DDH patients was reported to be 0.92%–2.93%^20^, ^21^, which was comparable to patients with other causes leading to primary THA. However, it is noteworthy that the postoperative dislocation rate in Crowe type IV hips was reported to be as high as 9.5%–15.0% in some follow‐up studies, with the sample size ranging from only 15 to 28 hips^22^, ^23^, ^24^, ^25^. Since the primary THA in Crowe type IV hips is technically challenging, it would be even more difficult to manage a recurrently dislocated THA in Crowe type IV hips. As a result, it is of vital importance to determine whether patients with Crowe type IV DDH are at higher risk of sustaining dislocation after THA than patients with Crowe type I, II, and III DDH. Furthermore, identifying the risk factors related to postoperative dislocation in Crowe type IV DDH patients is important. It helps to adjust reconstruction strategy, plan perioperative management, and predict risk stratification preoperatively. The ultimate goal of identifying related risk factors is decreasing the risk of postoperative dislocation in such patients.

To the best of our knowledge, there are no studies with a large sample size focusing on postoperative dislocation in Crowe type IV DDH patients and further identifying the risk factors for dislocation in such patients. The purpose of this study was to: (i) investigate the postoperative dislocation in Crowe type IV hips with a large sample size; (ii) determine whether Crowe type IV hips have a higher rate of postoperative dislocation when compared with Crowe type I, II, and III hips; and (iii) further identify the risk factors for dislocation in Crowe type IV DDH patients.

## Patients and Methods

### *Patient Selection*

The institutional review board of our hospital approved this study. All patients provided informed consent for participation. The inclusion criteria were: (i) patients were diagnosed as having Crowe type IV DDH; (ii) patients underwent THA in our institution from January 2009 to December 2017; (iii) each Crowe type IV hip was matched with three Crowe type I, II, or III hips according to gender, side and date of operation; (iv) patients should be able to provide information about postoperative dislocation; and (v) patients were retrospectively collected in the departmental database. The exclusion criteria were: (i) patients lost to follow‐up; and (ii) deceased. We reviewed 136 patients who were diagnosed as having Crowe type IV DDH and underwent THA from January 2009 to December 2017. A total of 25 patients were lost to follow‐up after surgery and six patients were deceased, with no deaths related to the THA procedure. Overall, 105 Crowe type IV DDH patients (131 hips) were included in this study. A total of 315 patients (393 hips) with Crowe type I, II, III DDH were identified as controls.

### *Surgical Technique*

#### *Anesthesia and Position*

All procedures were performed under general anesthesia with the patient in the lateral decubitus position.

#### *Approach and Exposure*

All procedures were performed via a posterolateral approach. For Crowe type IV DDH patients, resection of the hypertrophic capsule and femoral head was performed to expose the true acetabulum and proximal femur. The osteophytes and fibrous scar tissue were also removed.

#### *Pathological Changes and Preparation*

In Crowe type IV hips, small and shallow true acetabulum with anterior and superior bone deficiency, femoral neck with excessive anteversion, and horizontal orientation and weakened abductor muscle were often observed. The true acetabulum was gradually reamed with hemispherical reamers to reach the medial wall, with bleeding cancellous bone. In regard to the femur, reamers with gradual increase in size were used to prepare the femoral canal until the diaphyseal cortex was involved. If leg lengthening would be more than 4 cm after hip reduction, subtrochanteric shortening osteotomy was performed and extensive soft tissue release was avoided. Only tensor fascia lata, attachment of the gluteus maximus to the femoral crest, and attachment of the iliopsoas muscle to the lesser trochanter were released. We believe that subtrochanteric shortening osteotomy can maintain the soft‐tissue envelope and tension as much as possible and decrease the risk of excessively stretching the sciatic nerve.

#### *Placement of Prosthesis*

A porous‐coated acetabular component (Pinnacle, DePuy) at 15° ± 10° of anteversion and 40° ± 10° of inclination and a cementless modular femoral stem (S‐ROM, DePuy) at 15°–20° of anteversion were inserted in all Crowe type IV hips. A small acetabular prosthesis was often inserted and consequently a small femoral head was used.

#### *Postoperative Reconstruction*

Patients were encouraged to conduct early mobilization and limb exercises in bed immediately after surgery, especially hip abduction function exercise. They walked with partial weight bearing for approximately 2 weeks, and then gradually progressive full weight bearing was allowed at 4–6 weeks after surgery. Patients were followed up regularly after surgery.

### *Data Collection*

#### *Postoperative Dislocation*

All patients were asked to provide information about postoperative dislocation. They were contacted by telephone at the latest follow‐up and were asked specifically whether a postoperative dislocation had occurred. Information about dislocation that was treated at our institution was routinely documented and collected. Direction of dislocation was identified through cross‐table lateral radiographs or by reviewing clinical records, and interventions were confirmed by reviewing clinical records. Patients with dislocation that were treated at other institutions were asked to provide radiographs and clinical records.

In our department, the first dislocation after surgery was generally managed with closed or open reduction. When second dislocation was encountered, closed or open reduction combined with abduction bracing for 3 months were performed. In cases of recurrent dislocation that cannot be managed with non‐operative treatment, revision surgery would be contemplated and performed to correct the underlying etiology.

#### *Demographic and Implant Data*

Preoperative demographic data, including age, sex, BMI (body mass index), side, preoperative Trendelenburg sign, and preoperative range of motion, were documented from the clinical records in our hospital. A positive Trendelenburg test is one in which the pelvis drops on the contralateral side during a single‐leg stand on the affected side, which can often be found in DDH patients. A positive Trendelenburg sign usually indicates weakness in abductor muscles, such as gluteus medius and gluteus minimus. Implant data, including the length of femoral shortening osteotomy, femoral head size and acetabular size, were documented from the operation records.

#### *High Dislocation Type*

In Crowe type IV DDH patients, the type and severity of subluxation were determined by presence of false acetabulum and subluxation height. Measurements were made on preoperative and immediate postoperative AP pelvic radiographs. High dislocation in hips with or without a false acetabulum were distinguished according to Hartofilakidis^26^. The subluxation height was the distance from the head–neck junction to the interteardrop line.

#### *Limb Length*

Limb length, including limb‐length discrepancy and leg lengthening, were measured on preoperative and immediate postoperative AP pelvic radiographs of Crowe type IV DDH patients. Limb‐length discrepancy was measured as the difference in the vertical distance between the tip of the lesser trochanter and the interteardrop line between the operated and contralateral hips. Leg lengthening was calculated as follows, according to the formula previously described by Makita: leg lengthening = preoperative height of the greater trochanter ‐ postoperative height of the greater trochanter ‐ femoral shortening^27^. Restoration of lower limb length is the operative principles of THA. Lower limb length reflects the soft tissue tension around the hip and plays an important part in postoperative dislocation.

#### *Hip Offset*

Hip offset was measured on preoperative and immediate postoperative AP pelvic radiographs of Crowe type IV DDH patients. Femoral offset was the distance from the center of the femoral head to the anatomical femoral axis (center of medullary canal). Acetabular offset was measured as the horizontal distance from the center of the femoral head to the perpendicular line of the interteardrop line through the tip of the teardrop. Hip offset was the sum of the femoral offset and acetabulum offset^28^, ^29^. The discrepancy of hip offset was evaluated by comparing the operated and contralateral hips. Restoration of lower limb offset is one of the operative principles of THA. Hip offset reflects the soft tissue tension around the hip and plays an important part in postoperative dislocation.

#### *Cup Position*

**Figure 1:**
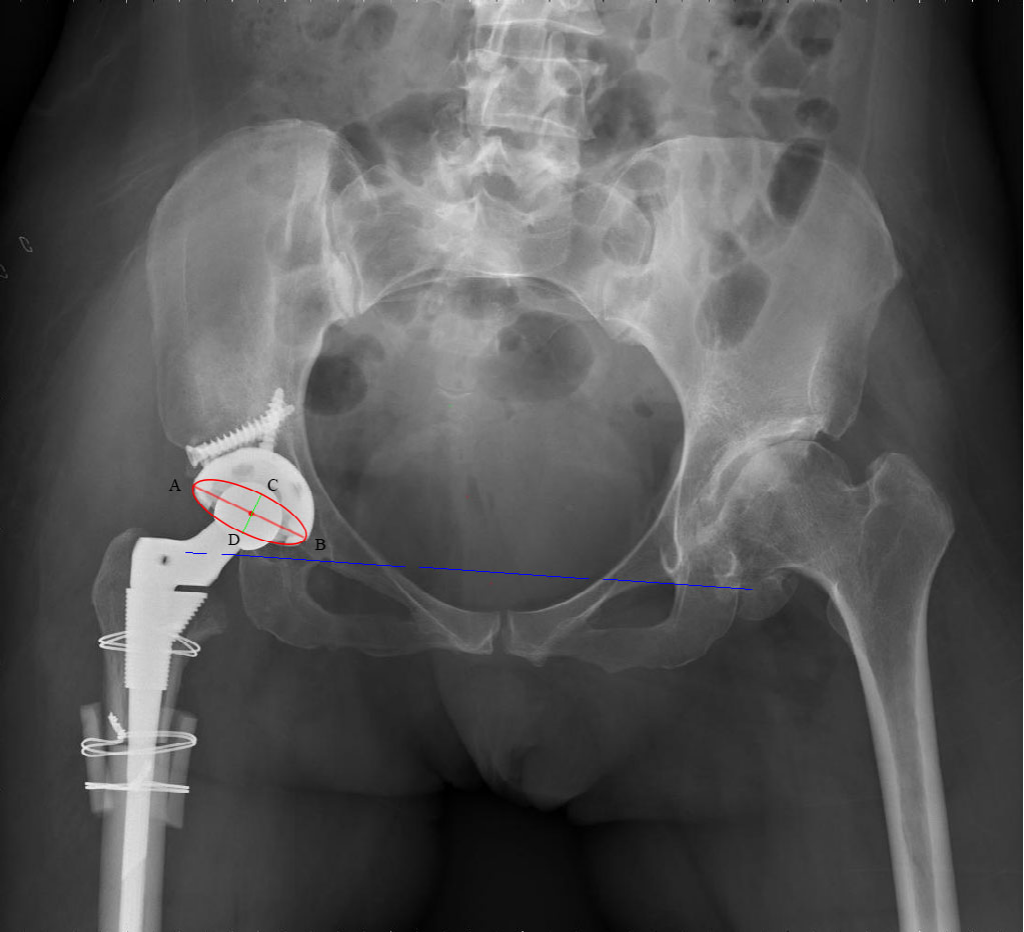
The acetabular orientation was measured on the AP pelvic radiograph. AB: long axis of the ellipse of the acetabular component. CD: short axis of the ellipse of the acetabular component. Acetabular anteversion = sin ^−1^ (CD/AB). Acetabular abduction is the angle between AB and interteardrop line (blue line).

The abduction and anteversion of the acetabular component were evaluated based on the AP pelvic radiograph, as described by Murray and others^30^, ^31^, ^32^. As shown in Fig.1, acetabular abduction is the angle between the long axis of the ellipse of the acetabular component and interteardrop line. Acetabular anteversion = sin ^−1^ (CD/AB) (AB: the long axis of the ellipse of the acetabular component; CD: short axis of the ellipse of the acetabular component). Cup position has an influence on the postoperative dislocation in Crowe type IV DDH patients.

### *Statistical Analysis*

Continuous variables, including age, BMI, preoperative range of motion, subluxation height, limb‐length discrepancy, leg lengthening, hip offset, abduction and anteversion of the acetabular component, and length of femoral shortening osteotomy, were presented as the mean ± standard deviation and were analyzed with two‐sided independent *t*‐test. Categorical variables, including Crowe type, sex, side, Trendelenburg sign, type of acetabulum, femoral head size, and acetabular size were presented as absolute value (percentage) and were analyzed with Pearson chi‐squared test. Univariate analyses were initially used in comparison between the Dislocation and No Dislocation groups in Crowe type IV hips. Variables which had *P* < 0.1 in the univariate analyses were included in the multivariate regression model. Multivariate logistic regression analysis was used to determine independent risk factors for dislocation in Crowe type IV hips. Analyses were performed with significance level α = 0.05, and all statistical analyses were performed with SPSS v22.0 (IBM, Armonk, NY, US).

## Result

### *Follow‐up*

A total of 131 Crowe type IV hips were followed up for a mean of 76.5 ± 28.1 months (range from 24.3 to 130.5). A total of 393 Crowe type I, II, and III hips, including 261 type I hips, 94 type II hips, and 38 type III hips, were identified as controls and followed up for a mean of 76.4 ± 28.2 months (range from 24.0 to 132.0). No significant difference was observed in follow‐up time between two groups (*P* = 0.804).

### *Postoperative Dislocation in 524 DDH Hips*

One or more dislocations occurred in 22 of the 524 dysplasia hips. The dislocation rate in the overall cohort of 524 dysplasia hips was 4.20%. The dislocation rates among Crowe type I, II, III and IV hips were 1.53% (4 out of 261), 2.13% (2 out of 94), 2.63% (1 out of 38), and 11.45% (15 out of 131), respectively. No significant difference was observed among type I, II, and III hips (Fisher's exact test, *P* = 0.574). We combined type I, II, and III hips, and compared them with type IV hips. Crowe type IV hips showed a significantly higher rate of dislocation than type I, II, and III hips (11.45% *vs* 1.78%, *P* < 0.001) (Table 1).

**Table 1:** Preoperative demographic data and implant factors of dysplasia hips

Indexes	Overall (*n* = 524)	No dislocation (*n* = 502)	Dislocation (*n* = 22)	*P* value
Age (years)	54.7 ± 14.7	54.8 ± 14.8	52.8 ± 12.1	0.539
Sex				0.463
Male	36 (6.9%)	35 (97.2%)	1 (2.8%)	
Female	488 (93.1%)	467 (95.7%)	21 (4.3%)	
BMI (kg/m^2^)	23.2 ± 3.7	23.2 ± 3.7	22.1 ± 2.9	0.173
Crowe classification				0.001
Types I, II, and III	393 (75.0%)	386 (98.2%)	7 (1.8%)	
Type IV	131 (25.0%)	116 (88.5%)	15 (11.5%)	
Unilateral/bilateral				0.744
Unilateral	316 (60.3%)	302 (95.6%)	14 (4.4%)	
Bilateral	208 (39.7%)	200 (96.2%)	8 (3.8%)	
Side				0.800
Left	252 (48.1%)	242 (96.0%)	10 (4.0%)	
Right	272 (51.9%)	260 (95.6%)	12 (4.4%)	
Preoperative Trendelenburg sign				0.027
Positive	396 (75.6%)	375 (94.7%)	21 (5.3%)	
Negative	128 (24.4%)	127 (99.2%)	1 (0.8%)	
Femoral head size (mm)				<0.001
22	30 (5.7%)	20 (66.7%)	10 (33.3%)	
28	139 (26.5%)	131 (94.3%)	8 (5.7%)	
32	248 (47.4%)	245 (98.8%)	3 (1.2%)	
≥36	107 (20.4%)	106 (99.1%)	1 (0.9%)	
Acetabular size (mm)				<0.001
≤44	121 (23.1%)	107 (88.4%)	14 (11.6%)	
46–48	271 (51.7%)	264 (97.4%)	7 (2.6%)	
≥50	132 (25.2%)	131 (99.3%)	1 (0.7%)	

BMI, body mass index; DDH, developmental dysplasia of the hip.

In the overall cohort of 524 dysplasia hips, the first dislocation of the 22 dislocated hips occurred at an average of 5.1 ± 14.4 months (range: 1 day to 60 months) postoperatively. A total of 19 dislocations (86.4%) occurred within 6 months, and these were considered early dislocation cases^9^. Posterior dislocation occurred in 12 hips (54.5%) and anterior dislocation occurred in five hips (22.7%). The direction of dislocation was not determined in five cases because of the lack of records.

Of the 22 dislocated hips, 20 hips (90.9%) were successfully managed with non‐operative treatment. A total of 17 hips (77.3%) with one dislocation were successfully treated with closed or open reduction, and three hips (13.6%) with more than one dislocation further had abduction bracing for 3 months. One patient (Crowe type I) experienced recurrent dislocation and underwent revision to a constrained acetabular component. Another patient (Crowe type IV) with mental disorder sustained recurrent dislocation 60 months after surgery due to aseptic loosening of the cup, but the patient refused revision surgery despite our advice.

### *Demographic and Implant Data in 524 DDH Hips*

We have compared the demographic data between the Dislocation and No Dislocation groups in the overall cohort of 524 dysplasia hips. Except for Crowe type, hips with preoperative positive Trendelenburg sign, smaller femoral head size, and smaller acetabular size showed a significantly higher rate of dislocation (Table 1). Hips with preoperative positive Trendelenburg sign had a significantly higher rate of dislocation than the hips with preoperative negative Trendelenburg sign (5.3% *vs* 0.8%, *P* = 0.027). The size of femoral head was related to dislocation rate (*P* < 0.001): 22‐mm femoral head with a dislocation rate of 33.3% (10 cases); 28‐mm femoral head with a dislocation rate of 5.7% (eight cases); 32‐mm femoral head with a dislocation rate of 1.2% (three cases); over 32‐mm femoral head with a dislocation rate of 0.9% (one case). The acetabular size was also related to dislocation rate (*P* < 0.001): below 46‐mm acetabular cup with a dislocation rate of 11.6% (14 cases); 46 to 48‐mm acetabular cup with a dislocation rate of 2.6% (seven cases); over 48‐mm femoral head with a dislocation rate of 0.7% (one case). No significant difference was observed between the Dislocation and No Dislocation groups in age, sex, BMI and operation side.

### *Postoperative Dislocation in Type IV Hips*

**Figure 2:**
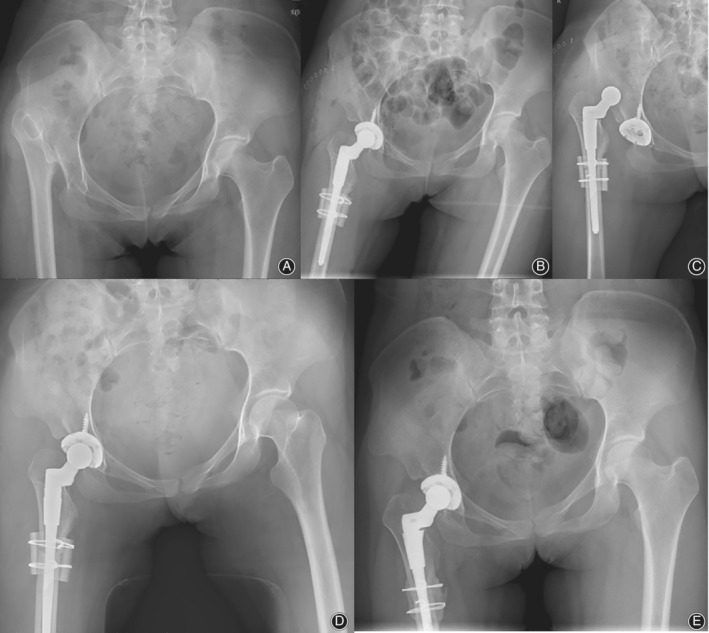
Radiographs of a 56‐year‐old woman with unilateral Crowe type IV DDH. (A) preoperative radiographic image showing high dislocation of the right hip without a false acetabulum. (B) Radiographic image at post‐operative day 1 showing that THA with a 22‐mm metal femoral head was performed in the right hip. (C) Dislocation occurred at post‐operative day 5. (D) Closed reduction was performed. (E) At the 4‐year follow‐up, no further dislocation or loosening of the component occurred.

**Figure 3:**
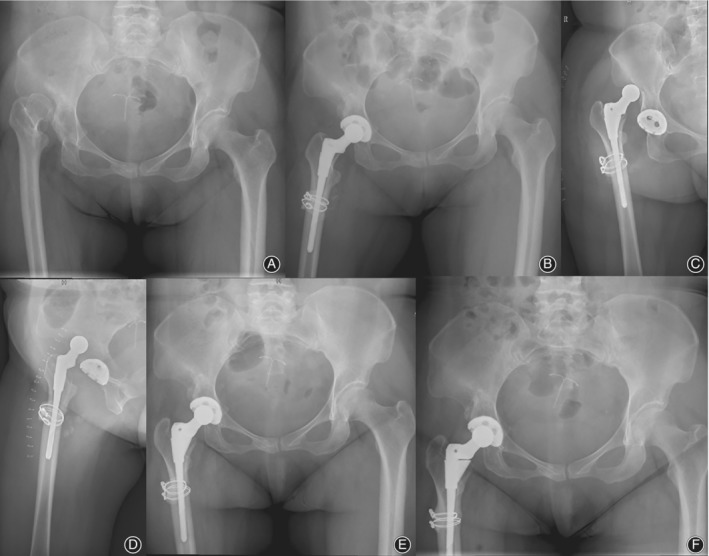
Radiographs of a 59‐year‐old woman with unilateral Crowe type IV DDH. (A) preoperative radiographic image showing high dislocation of the right hip. (B) Radiographic image at post‐operative day 1 showing that THA with a 22‐mm metal femoral head was performed in the right hip. (C, D) Dislocation occurred at post‐operative day 2. (E) Closed reduction was performed. (F) At the 5‐year follow‐up, no further dislocation or loosening of the component was identified.

**Figure 4:**
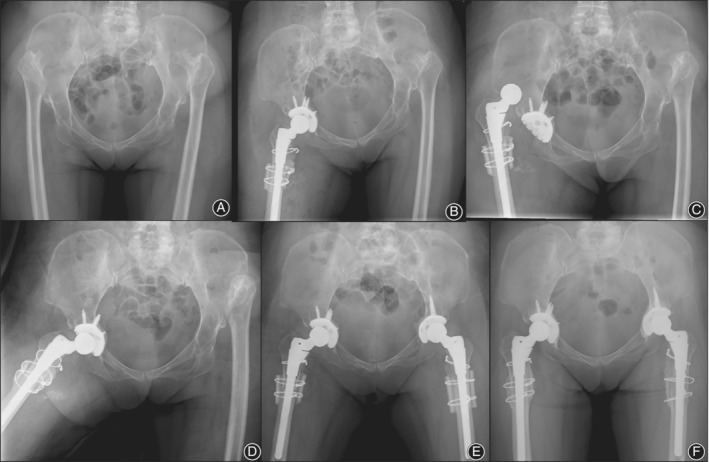
Radiographs of a 66‐year‐old woman with bilateral Crowe type IV DDH. (A) preoperative radiographic image showing high dislocation of both hips without a false acetabulum. (B) Radiographic image at post‐operation day 1. THA with 28‐mm metal femoral head performed in the right hip. (C) Dislocation occurred at post‐operation day 9. (D) Closed reduction was performed followed by abduction bracing for 3 months. (E) Postoperative radiographic image showing THA combined with transverse osteotomy in the left hip. (F) At 8‐year follow‐up, no more dislocation occurred and no loosening of components was identified.

One or more dislocations occurred in 15 hips of the 131 Crowe type IV hips. (Figs 2, 3, 4) The dislocation rate was 11.45%. The first dislocation occurred at an average of 5.2 ± 15.5 months. A total of 13 dislocations (86.7%) occurred within 6 months, and these were considered early dislocation cases. Posterior dislocation occurred in nine hips (60%) and anterior dislocation occurred in three hips (20%). The direction of the three dislocations was not determined because of the lack of records. A total of 11 hips with one dislocation were successfully treated with closed reduction, and three hips with more than one dislocation further had abduction bracing for 3 months. All dislocations, except one (mentioned above), were successfully managed with non‐operative treatment.

### *Demographic and Implant Data in Type IV Hips*

Demographic and implant data of Crowe type IV hips were compared between the Dislocation and No Dislocation groups (Table 2). Dislocation cases had significantly older age than no dislocation cases (51.0 ± 13.2 *vs* 44.7 ± 10.8, *P* = 0.04). Dislocation cases had significantly lower preoperative flexion range of motion than no dislocation cases (87.1 ± 21.8 *vs* 95.7 ± 17.9, *P* = 0.037). The size of femoral head was related to dislocation rate (*P* < 0.001): 22‐mm femoral head with a dislocation rate of 34.6% (nine cases); 28‐mm femoral head with a dislocation rate of 6.3% (five cases); over 28‐mm femoral head with a dislocation rate of 3.8% (one case). No significant difference was observed between the Dislocation and No Dislocation groups in sex, BMI, operation side, preoperative extension range of motion, abduction, adduction, external rotation, internal rotation, length of femoral shortening osteotomy, and acetabular size. All Crowe type IV hips showed preoperative positive Trendelenburg sign.

**Table 2:** Demographic, radiographic, and implant data of Crowe type IV dysplasia hips

Indexes	Overall (*n* = 131)	No dislocation (*n* = 116)	Dislocation (*n* = 15)	*P* value
Age (years)	45.4 ± 11.2	44.7 ± 10.8	51.0 ± 13.2	0.04*
Sex				0.254
Male	9 (6.9%)	9 (100%)	0 (0%)	
Female	122 (93.1%)	107 (87.7%)	15 (12.3%)	
BMI (kg/m^2^)	22.2 ± 3.6	22.3 ± 3.7	21.1 ± 2.8	0.076*
Unilateral/bilateral				0.593
Unilateral	79 (60.3%)	69 (87.3%)	10 (12.7%)	
Bilateral	52 (39.7%)	47 (90.4%)	5 (9.6%)	
Side				0.907
Left	63 (48.1%)	56 (88.9%)	7 (11.1%)	
Right	68 (51.9%)	60 (88.2%)	8 (11.8%)	
Preoperative Trendelenburg sign				NA
Positive	131 (100%)	116 (88.5%)	15 (11.5%)	
Negative	0 (0%)	0 (0%)	0 (0%)	
False acetabulum				< 0.001*
Absence	60 (45.8%)	47 (78.3%)	13 (21.7%)	
Presence	71 (54.2%)	69 (97.2%)	2 (2.8%)	
Preoperative range of motion (°)				
Flexion	88.0 ± 21.1	87.1 ± 21.8	95.7 ± 17.9	0.037*
Extension	1.5 ± 4.0	1.6 ± 4.1	1.3 ± 3.9	0.844
Abduction	23.8 ± 11.9	23.7 ± 12.2	25.1 ± 10.5	0.655
Adduction	21.0 ± 12.8	20.8 ± 13.1	22.3 ± 11.1	0.678
External rotation	19.3 ± 16.2	19.1 ± 16.5	21.3 ± 13.9	0.624
Internal rotation	16.4 ± 13.3	16.1 ± 13.5	18.7 ± 11.4	0.487
Subluxation height (mm)	68.9 ± 20.3	69.0 ± 20.4	68.1 ± 19.3	0.868
Preoperative limb‐length discrepancy (mm)	−22.7 ± 50.2	−22.8 ± 49.4	−21.2 ± 58.7	0.659
Postoperative limb‐length discrepancy (mm)	4.9 ± 18.1	4.7 ± 18.5	6.3 ± 17.6	0.401
Length of femoral shortening osteotomy (mm)	34.8 ± 13.5	34.6 ± 14.6	36.4 ± 9.0	0.514
Leg lengthening (mm)	33.8 ± 25.6	33.6 ± 27.4	34.2 ± 10.3	0.865
Preoperative hip offset (mm)	−3.1 ± 9.4	−3.3 ± 9.5	−0.7 ± 8.1	0.527
Postoperative hip offset (mm)	−3.0 ± 8.5	−3.1 ± 8.6	−2.6 ± 7.6	0.268
Cup inclination (°)	38.9 ± 9.5	38.7 ± 9.5	40.6 ± 8.5	0.134
Cup anteversion (°)	19.8 ± 4.0	20.0 ± 4.1	18.1 ± 3.2	0.081*
Femoral head size (mm)				<0.001*
22	26 (19.8%)	17 (65.4%)	9 (34.6%)	
28	79 (60.2%)	74 (93.7%)	5 (6.3%)	
≥32	26 (19.8%)	25 (96.2%)	1 (3.8%)	
Acetabular size (mm)				0.476
≤44	80 (61.1%)	69 (86.3%)	11 (13.7%)	
46–48	45 (34.4%)	41 (91.1%)	4 (8.9%)	
≥50	6 (4.6%)	6 (100%)	0 (0%)	

* Variables with *P* < 0.1 were included in multivariate logistic regression analysis.

### *High Dislocation Type in Type IV Hips*

Hips without false acetabulum had significantly higher rate of dislocation than hips with false acetabulum (21.7% *vs* 2.8%, *P* < 0.001). No significant difference was observed in subluxation height between the Dislocation and No Dislocation groups (68.1 ± 19.3 *vs* 69.0 ± 20.4, respectively; *P* = 0.868).

### *Limb Length in Type IV Hips*

No significant difference was observed in preoperative limb‐length discrepancy between the Dislocation and No Dislocation groups (−21.2 ± 58.7 *vs* −22.8 ± 49.4, respectively; *P* = 0.659). No significant difference was observed in postoperative limb‐length discrepancy between the Dislocation and No Dislocation groups (6.3 ± 17.6 *vs* 4.7 ± 18.5, respectively; *P* = 0.401). No significant difference was observed in leg lengthening between the Dislocation and No Dislocation groups (34.2 ± 10.3 *vs* 33.6 ± 27.4, respectively; *P* = 0.865).

### *Hip Offset in Type IV Hips*

No significant difference was observed in preoperative hip offset between the Dislocation and No Dislocation groups (−0.7 ± 8.1 *vs* −3.3 ± 9.5, respectively; *P* = 0.527). No significant difference was observed in postoperative hip offset between the Dislocation and No Dislocation groups (−2.6 ± 7.6 *vs* −3.1 ± 8.6, respectively; *P* = 0.268).

### *Cup Position in Type IV Hips*

No significant difference was observed in cup inclination between the Dislocation and No Dislocation groups (40.6 ± 8.5 *vs* 38.7 ± 9.5, respectively; *P* = 0.134). No significant difference was observed in cup anteversion between the Dislocation and No Dislocation groups (18.1 ± 3.2 *vs* 20.0 ± 4.1, respectively; *P* = 0.081).

### *Multivariate Logistic Regression Analysis*

Variables showing *P* < 0.1 between the Dislocation and No Dislocation groups in the univariate analyses, including age, BMI, presence of a false acetabulum, preoperative flexion range of motion, cup anteversion and femoral head size, were included in the multivariate regression model (Table 2). The use of a 22‐mm femoral head when compared with the use of a femoral head >32 mm (odds ratio [*OR*] = 23.55, 95% confidence interval [*CI*] = 1.901–291.788, *P* = 0.014), older age (*OR* = 1.128, 95% *CI* = 1.037–1.275, *P* = 0.031), and absence of false acetabulum (*OR* = 12.425, 95% *CI *= 1.982–77.879, *P* = 0.007) were identified as independent risk factors for dislocation in Crowe type IV hips (Table 3). BMI, preoperative flexion range of motion, and cup anteversion were not identified as independent risk factors for dislocation in Crowe type IV hips in this multivariate model after adjusting for other factors, and were determined to be confounding variables.

**Table 3:** Multivariate logistic regression analysis identifying risk factors for dislocation in Crowe type IV DDH patients

Variables	Odds ratio	95% confidence interval	*P* value
Femoral head size (mm)			<0.001
22*	23.55	1.901‐291.788	0.014
28	1.59	0.147–17.282	0.7
≥32 (reference)	1	‐	‐
Age*	1.128	1.037‐1.275	0.031
BMI	0.943	0.768–1.158	0.577
Absence of false acetabulum*	12.425	1.982–77.879	0.007
Flexion	1	0.960–1.041	1
Cup inclination	0.878	0.72–1.071	0.2

BMI, body mass index; DDH, developmental dysplasia of the hip.

* Showing statistical significance in multivariate model.

## Discussion

### *High Risk of Dislocation in Type IV Hips*

A few follow‐up studies with a small sample size investigating Crowe type IV hips found that the dislocation rates after THA can be as high as 9.5%–15.0%^22^, ^23^, ^24^, ^25^. To the best of our knowledge, there are no studies with a large sample size focusing on postoperative dislocation in Crowe type IV DDH patients. The sample size of Crowe type IV hips in our study was large (131 hips) with a control group of 393 type I, II, or III hips, giving it considerable power to detect the difference between two groups. We found that 15 postoperative dislocations occurred in 131 Crowe type IV dysplasia hips, with a dislocation rate of 11.45%, which was significantly higher than that observed in Crowe type I, II, or III hips (1.78%, *P* < 0.001).

### *Risk Factors: Small Femoral Head*

We further investigated the risk factors for dislocation in Crowe type IV DDH patients. The use of a 22‐mm femoral head was identified as a risk factor for dislocation in Crowe type IV DDH patients. We found that when compared with the 32‐mm femoral head, the use of a 28‐mm femoral head did not increase the dislocation rate and the use of a 22‐mm femoral head increased the risk. This is in contrast to the findings of Wang who found that 22‐mm and 28‐mm femoral heads were both risk factors for dislocation in DDH patients^20^. The small femoral head diameter has been widely accepted as a risk factor for dislocation after THA because of increased component impingement and decreased “jump distance”^7^, ^8^. In THA of Crowe type IV hips, the general consensus is that placement of acetabular component in the true acetabulum instead of a high placement can provide better hip function and longer prosthetic durability^33^, ^34^. As a result, we placed the cup in the true acetabulum, which was hypoplastic, shallow, and bone deficient. However, to achieve >70% cup coverage, only a small acetabular prosthesis can be inserted and consequently a small femoral head was used, which explained the high rate of dislocation in Crowe type IV hips.

### *Risk Factors: Old Age*

Older age was identified as a risk factor for dislocation after primary THA^6^, ^35^, and this was also confirmed in the Crowe type IV DDH population in our study. This phenomenon may be due to abductor muscle weakness, which is the most important recognized risk factor for hip instability after THA and clinically associated with a positive Trendelenburg sign^9^. In fact, radiographic geometric analysis found that the area, length, and strength of the gluteus medius muscle significantly decreased in DDH patients^15^, especially in hips with high dislocation^36^. Older age may further decrease the strength of the abductor muscle in Crowe type IV DDH patients, who had an already weak abductor muscle, and predispose them to a higher risk of dislocation. Most of the dislocations in Crowe type IV DDH patients occurred within 6 months after THA in our study, which can be explained by the fact that the abductor strength improved significantly after 6 months postoperatively in DDH patients^9^, ^36^.

### *Risk Factors: False Acetabulum*

We found that the high dislocation hips without preoperative false acetabulum formation had a 12 times higher risk of postoperative dislocation than the hips with false acetabulum. This is in accordance with the findings of Hartofilakidis *et al*.; although they did not pay attention to the difference in the dislocation rates between different types of high dislocation^26^. In Hartofilakidis' study, three dislocations occurred out of 30 hips in the no false acetabulum group (10%) while there were zero out of 49 hips in the false acetabulum group (0%)^26^. This can be explained by the fact hips without false acetabulum have a worse soft tissue condition and abductor muscle strength because of a high‐riding femoral head in the gluteal musculature, and it has a more abnormal proximal femur shape caused by the lower mechanical loads^37^.

### *Soft Tissue Tension*

In our experience, the soft tissue imbalance may also play a role in postoperative hip instability in DDH patients with high dislocation. After reduction of components, soft tissue tension may be inadequate in the gluteus medius, gluteus minimus, and piriformis muscles, whereas excessive soft tissue tension can be found in the tensor fascia lata and biceps femoris muscle. In addition, valgus inclination of the lower limb after THA caused by excessive soft tissue tension in the iliotibial tract predisposed Crowe type IV DDH patients to a hip adduction and internal rotation position, which increased the risk of posterior dislocation.

### *Safe Zone*

One long‐held tenet is that cup position in a “safe zone” of 15 ± 10 degrees of anteversion and 40 ± 10 degrees of inclination, described by Lewinnek *et al*., can minimize the postoperative dislocation occurrence^12^. However, it has been reported that “safe zone” alone is not protective against instability and the ideal cup position for some high‐risk patients lies outside the Lewinnek “safe zone”^31^, ^38^. This was confirmed by our study: cup position in most dislocation cases lied inside the “safe zone” and no difference in cup position was observed between the Dislocation and No Dislocation groups in Crowe type IV patients. The ideal cup position for Crowe type IV DDH patients requires further investigation.

### *Suggestions for Reducing Dislocation*

Based on our findings, we suggest large femoral heads should be utilized to provide better hip stability in Crowe type IV patients. Increasing the femoral head size from 22 mm to 28 mm can be reliable and effective to decrease the dislocation rate. Surgeons may choose other approaches to increase the cup coverage, including the medial protrusio technique, use of autologous bulk bone graft and augment, and avoid excessive decrease of the diameter of the acetabulum and femoral head^39^, ^40^. Close attention – including enhanced preoperative patient education, postoperative precaution, and prohibited hip positions and maneuvers – should be paid to Crowe type IV patients, especially older patients and patients without a false acetabulum.

Furthermore, we believe that preoperative and postoperative improvement in abductor muscle strength plays an important role in preventing postoperative dislocation in Crowe type IV patients, since it has been reported that an extended rehabilitation program for strengthening the gluteal muscles in DDH patients after THA can be effective^41^. However, the rehabilitation protocol for Crowe type IV DDH patients needs to be further investigated. Although early rehabilitation can provide better long‐term hip function for THA patients, excessive exercise may also exacerbate soft tissue laxity and hip instability^42^.

Complete resection of the hypertrophic capsule was usually performed in Crowe type IV dysplasia hips to expose the true acetabulum and surrounding structure in our study. This was particularly important for patients with previous hip osteotomy because the scar tissue added difficulty to the THA surgery. However, one cadaver study found that the posterior capsule significantly contributes to hip stability^43^. We speculate that preservation of the posterior capsule actually has no influence on the intraoperative exposure and reduction, and maintains the soft‐tissue envelope as much as possible, which may be an effective way to increase the stability of the hip in Crowe type IV dysplasia hips.

### *Limitations*

There are several limitations to our study. First, this is a retrospective study with an inevitable bias in patient selection. Second, it is more accurate to determine the acetabular component position with computed tomography than with AP radiography. However, we believed that the radiographs were sufficient because we were comparing relative positions between the Dislocation and No Dislocation groups. Third, we were unable to evaluate the stem version and combined anteversion because cross‐table radiography and computed tomography were not performed routinely after surgery. Further studies with more accurate measurements and longer follow‐up time are required to confirm the findings of this study.

### *Conclusion*

Crowe type IV DDH patients had a high risk of dislocation after THA. Increasing the femoral head size from 22 mm to 28 mm can reliably and effectively decrease the dislocation rate in these patients. Special attention should be paid to older patients and those without a false acetabulum. Besides, improving abductor muscle strength through preoperative and postoperative rehabilitation programs may help decrease the rate of postoperative dislocation in Crowe type IV DDH patients. Closed reduction combined with abduction bracing can be effective to manage postoperative dislocation in these patients.
